# Association of Single-Nucleotide Polymorphisms of rs2383206, rs2383207, and rs10757278 With Stroke Risk in the Chinese Population: A Meta-analysis

**DOI:** 10.3389/fgene.2022.905619

**Published:** 2022-06-28

**Authors:** Xuemei Hu, Dongsen Wang, Chunying Cui, Qingjian Wu

**Affiliations:** ^1^ Clinical Medical College of Jining Medical University, Jining, China; ^2^ Department of Emergency, Jining No. 1 People’s Hospital, Jining, China

**Keywords:** ischemic stroke, chromosome 9p21, rs2383206, rs2383207, rs10757278, Chinese

## Abstract

Several studies have reported that chromosome 9p21 is significantly associated with ischemic stroke (IS) risk, with the G allele associated with increased risk. However, controversial results have been reported in the literature. We systematically assessed the relationship between stroke and three 9p21 loci (rs2383206, rs2383207, and rs10757278) in this meta-analysis. First, we searched the PubMed and Embase databases for relevant studies. We then calculated odds ratios using the chi-squared test. The evaluation of experimental data was performed using bias tests and sensitivity analyses. We analyzed data from 16 studies involving 18,584 individuals of Chinese ancestry, including 14,033 cases and 14,656 controls. Our results indicated that chromosome 9p21 is significantly associated with IS (odds ratio: 1.15, 95% confidence interval: 1.1–1.20, *p* < 0.0001). Because the three single-nucleotide polymorphisms (rs2383206, rs2383207, and 10757278) have a linkage disequilibrium relationship, all three may increase the risk of IS.

## Introduction

Stroke is a severe disease and is the leading cause of disability and death in China (Liu et al., 2011). It is an acute cerebrovascular disease that is characterized by focal loss of nerve function and high mortality and disability, and it currently poses a serious threat to human life and health (Li et al., 2021). Stroke is thought to be caused by environmental risk factors, multiple genes, and their interactions. To date, however, a large proportion of stroke risk remains unexplained (Ganesh et al., 2016). Genetic variation on chromosome 9p21 is widely believed to be linked to risk of coronary heart disease (McPherson et al., 2007; Samani et al., 2007), but it has a different role in stroke (Matarin et al., 2008; Gschwendtner et al., 2009). Previously, genome-wide association studies (GWAS) have analyzed genes associated with ischemic stroke (IS) (Söderholm et al., 2019). Single-nucleotide polymorphisms (SNPs) of rs2383206, rs2383207, and rs10757278 on chromosome 9p21 are linked to stroke. However, although several recent genetic studies have reported that chromosome 9p21 plays an important role in the mechanism of stroke, studies of different races and from different geographic locations have provided very different results. Therefore, an association between 9p21 polymorphisms and stroke has been established for individuals of European descent; the main aim of this meta-analysis was to study the relationship between three SNPs on chromosome 9p21 and stroke in the Chinese population.

## Methods

### Search Strategy

We searched the PubMed and Embase databases and selected all possible studies using the keywords “Stroke Chinese” and “rs2383206,” “rs2383207,” “rs10757278,” and “9p21.” The relevant literature was updated on 31 January 2022.

### Selection Criteria

The following selection criteria were used: (1) an association between the proposed SNPs and stroke was evaluated using a case–control design; (2) an accurate genotype number was provided or could be calculated (Liu et al., 2013); (3) the odds ratio (OR) and 95% confidence interval (CI) were provided to measure the risk of disease; (4) the OR value and 95% CI were calculated by providing enough data; (5) the same diagnostic criteria were used for stroke. The exclusion criteria were (1) the research was presented as a poster presentation, summary, meta-analysis, conference summary or article, or case series analysis; (2) the study was not performed in a Chinese population; (3) the three SNPs were not used; (4) the study was not consistent with the research topic; and (5) the exact number of genotypes was not provided and could not be calculated and/or the OR and 95% CI were not provided and could not be calculated. Two authors (DW and XH) independently screened all studies by their title or abstract and then evaluated the full text. Any differences in opinion were resolved through discussion.

### Data Extraction

Trial data from each identified study were extracted separately by two investigators (DW and XH). Any differences were eliminated by discussing the data extraction for each study using standard data collection tables. The data and information that were extracted for inclusion in the analysis included the first author’s name, publication year, language, population, study type, sample size, numbers, and frequencies of rs2383206, rs2383207, and rs10757278 polymorphism genotypes in the cases and controls, ORs, and 95% CIs. All extracted data are presented in Tables 1, 2.

**TABLE 1:** Sixteen studies in 11 articles investigating the association between rs2383207, rs2383206, and rs10757278 and IS.

SNP	First author; year	Population	Case	Control	Case genotype			Control genotype		
					GG	GA	AA	GG	GA	AA
rs2383207	Lin-2011	Chinese	627	1,349	288	274	65	568	609	172
	Jin-2021	Chinese	1,640	1755	795	665	180	815	690	250
	Yang-2018	Chinese	550	548	236	237	77	244	251	53
	Li-2017	Chinese	1,429	1,191	633	642	154	492	525	174
	Li-2021^1^	Chinese	987	946	480	425	82	410	407	129
	Zhang-2012^2^	Chinese	1,657	1,664	700	743	214	652	796	216
rs2303206	Hua-2009	Chinese	352	423	67	188	97	78	191	154
	Ding-2009^4^	Chinese	440	498	113	213	114	94	264	140
	Li-2021^1^	Chinese	1,006	949	233	493	280	197	447	305
	Xiong-2018^3^	Chinese	200	205	48	96	56	46	98	61
	Zhang-2012^2^	Chinese	1,657	1,664	379	802	476	317	833	514
rs10757278	Bi-2015	Chinese	116	118	29	49	38	15	47	56
	Han-2020	Chinese	505	652	149	235	121	140	310	203
	Xiong-2018^3^	Chinese	200	205	52	95	53	47	99	59
	Ding-2009^4^	Chinese	441	501	40	181	220	45	236	220
	Zhang-2021^2^	Chinese	1,657	1,664	509	774	374	420	832	412

Note: The same numbers indicate the same article. SNP, single-nucleotide polymorphism.

**TABLE 2:** Correlation analysis between different genetic patterns of rs2383207, rs2383206, and rs10757278 at 9p21 locus and IS susceptibility.

SNP	First author; year	G (case/control)	A (case/control)	OR	95% CI	SE (ln (OR))
rs2383207	Lin-2011	850/1745	404/953	1.15	0.997 ∼ 1.325	0.073
	Jin-2021	2255/2320	1,025/1,190	1.13	1.019 ∼ 1.249	0.052
	Yang-2018	709/391	739/357	0.88	0.734 ∼ 1.045	0.09
	Li-2017	1908/1,509	950/873	1.16	1.037 ∼ 1.302	0.058
	Li-2021^1^	1,385/1,227	589/665	1.27	1.114 ∼ 1.458	0.069
	Zhang-2012^2^	2143/2100	1,171/1,228	1.07	0.968 ∼ 1.183	0.051
rs2303206	Hua-2009	322/347	382/499	1.21	0.988 ∼ 1.479	0.103
	Ding-2009^4^	439/452	441/544	1.19	0.999 ∼ 1.437	0.093
	Li-2021^1^	959/841	1,053/1,057	1.14	1.009 ∼ 1.298	0.064
	Xiong-2018^3^	192/190	208/220	1.07	0.811 ∼ 1.408	0.141
	Zhang-2012^2^	1,560/1,467	1754/1801	1.12	1.024 ∼ 1.243	0.049
rs10757278	Bi-2015	107/77	125/159	1.75	1.199 ∼ 2.541	0.192
	Han-2020	533/590	477/714	1.35	1.147 ∼ 1.594	0.084
	Xiong-2018^3^	199/193	201/217	1.11	0.845 ∼ 1.467	0.141
	Ding-2009^4^	261/326	621/676	0.87	0.716 ∼ 1.060	0.100
	Zhang-2021^2^	1792/1,672	1,522/1,656	1.17	1.059 ∼ 1.284	0.049

Note: The same numbers indicate the same article; SNP, single-nucleotide polymorphism; OR, odds ratio; CI, confidence interval; SE, standard error.

### Statistical Analysis

We investigated the Hardy–Weinberg equilibrium of rs2383206, rs2383207, and rs10757278. We also investigated their association with stroke using the chi-squared test, which was performed using R (http://www.r-project.org/) (Liu et al., 2013). For the meta-analysis, we determined the heterogeneity among datasets using Cochran’s *Q* test and *I* ^*2*^ = ^*(*^Q – (k – 1^*))*^ */*_*Q*_ × 100%. The *Q* statistic approximately follows a χ2 distribution, with *k-*1 degrees of freedom (*k* is the number of studies in the analysis) (Liu et al., 2017). When *I* ^*2*^ was greater than 50% and the *p*-value was less than 0.1 (Higgins et al., 2021), the DerSimonian and Laird random-effects model was used as the pooling method; otherwise, the Mantel–Haenszel or inverse variance fixed-effects model was used as the pooling method, as appropriate. We also used funnel plots to assess potential publication bias. When there is no bias, funnel plots are symmetrical; conversely, when bias is present, funnel plots are asymmetrical (Liu et al., 2014).

## Results

### Characteristics of Included Studies

**FIGURE 1:**
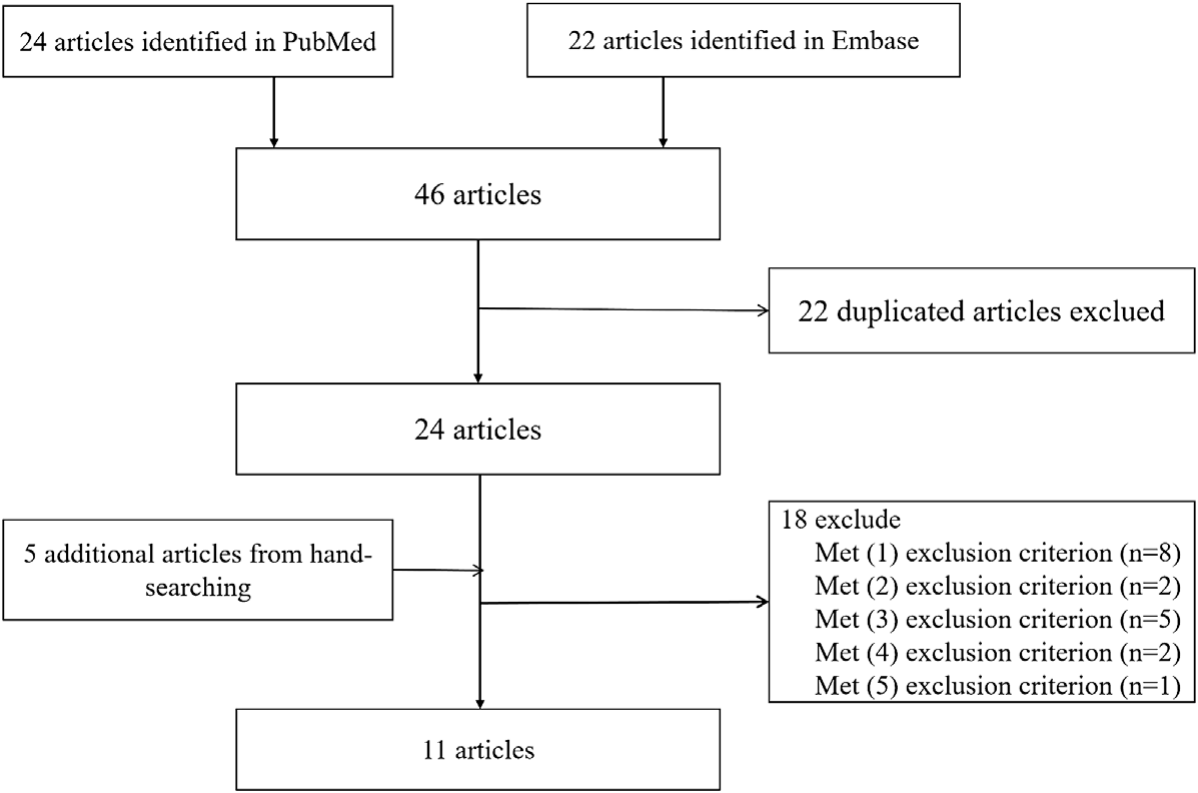
Flow chart of study selection in this meta-analysis.

In this meta-analysis, 18,584 participants were included: 14,033 in the IS group (7,235 cases with rs2383207, 3,762 cases with rs2383206, and 3,036 cases with rs10757278) and 14,656 cases in the control group (7,653 cases with rs2383207, 3,807 cases with rs2383206, and 3,196 cases with rs10757278). Eleven articles were selected, comprising 16 studies, of which six investigated rs2383207 (Lin et al., 2011; Zhang et al., 2012; Li et al., 2017; Yang et al., 2018; Jin et al., 2021; Li et al., 2021), five investigated rs2383206 (Ding et al., 2009; Hu et al., 2009; Zhang et al., 2012; Xiong et al., 2018; Li et al., 2021), and five investigated rs10757278 (Ding et al., 2009; Zhang et al., 2012; Bi et al., 2015; Xiong et al., 2018; Han et al., 2020). The study identification and selection process is shown in detail in Figure 1.

### Linkage Disequilibrium

The three SNPs—rs10757278, rs2383206, and rs2383207—are located within 10 kb of one another on chromosome 9p21 (https://snipa.helmholtz-muenchen.de/snipa3/).

### Meta-Analysis Results of 9p21

**FIGURE 2:**
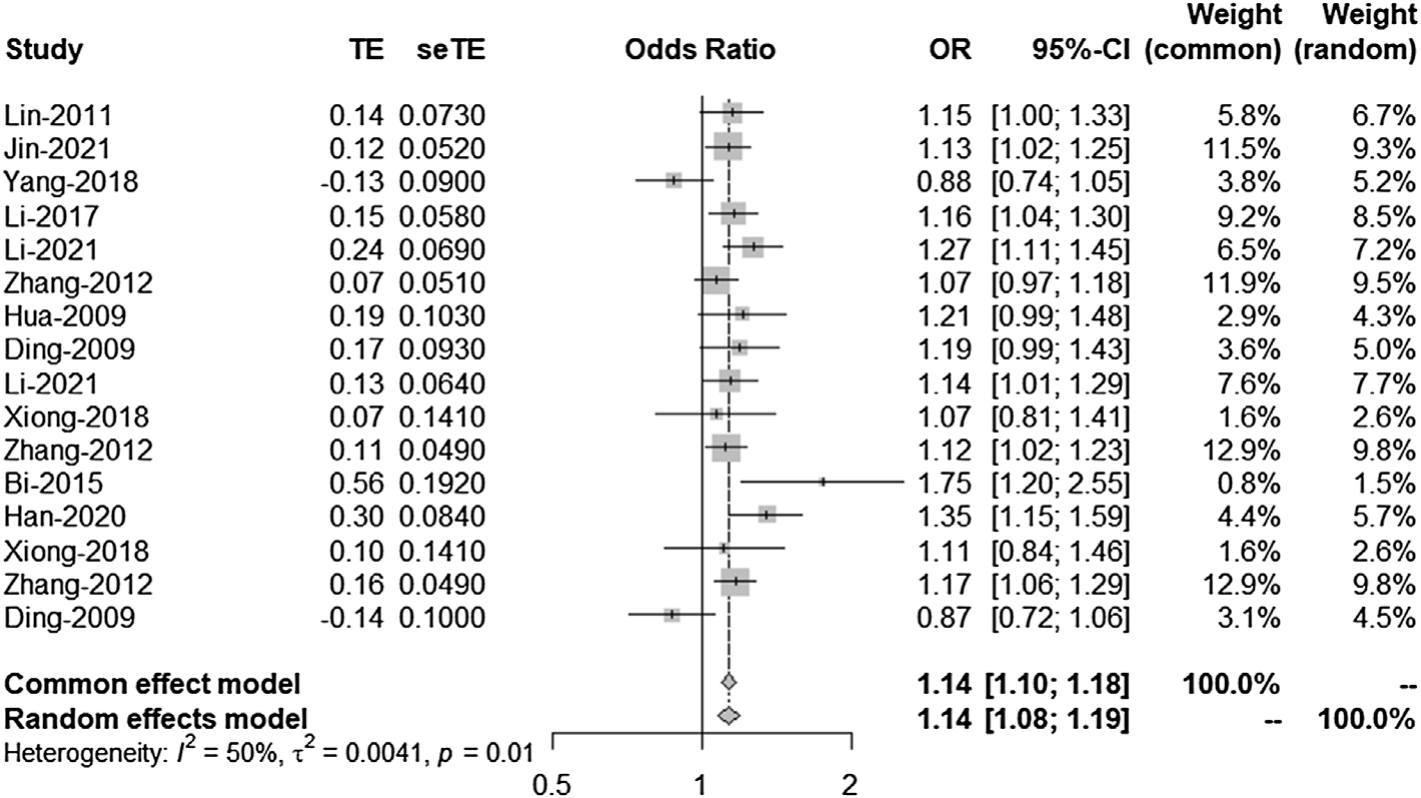
Random-effects meta-analysis of the association between the three single-nucleotide polymorphisms (SNPs; rs2383207, rs2383206, and rs10757278) and ischemic stroke (IS). CI, confidence interval; OR, odds ratios.

There is a linkage disequilibrium relationship among the three SNPS (rs2303206, rs2383207, and rs10757278). Thus, we performed an analysis of the OR values of all studies involving rs2383206, rs2383207, and rs10757278 in which the G allele was a minor allele. Because *I* ^2^ = 50%, a random-effects model was used to compare alleles (Figure 2). Chromosome 9p21 was significantly associated with IS risk, and the G allele was associated with increased IS risk (OR: 1.14, 95% CI: 1.08–1.19, *p* < 0.0001, Figure 2).

### Publication Bias

**FIGURE 3:**
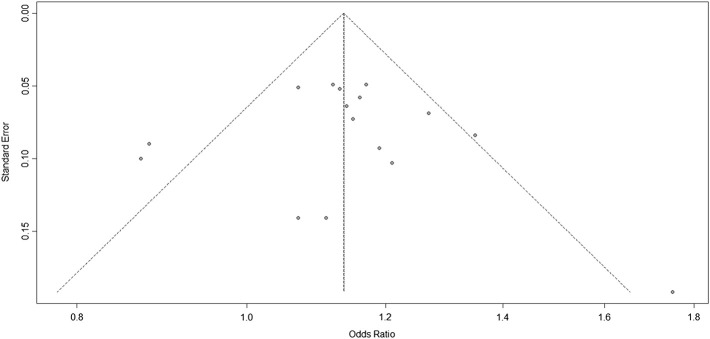
Funnel plots corresponding to the random-effects meta-analysis of all studies of the three single-nucleotide polymorphisms (SNPs; rs2383207, rs2383206, and rs10757278) and ischemic stroke (IS).

The Harbord test was used to evaluate publication bias. The bias = 0.3228, *p* = 0.7661, indicating no publication bias in the studies of 9p21 (Figure 3).

### Sensitivity Analysis

Because the *I* ^*2*^ is > 50% in this meta-analysis, a random-effects model was used. To assess the impact of each individual study on the pooled effect estimate, we performed a sensitivity analysis by removing one study at a time. The pooled estimate *I* ^2^ = 49.8%; thus, no single study significantly affected the results of each single-locus sensitivity analysis.

### Second and Third Analyses

**FIGURE 4:**
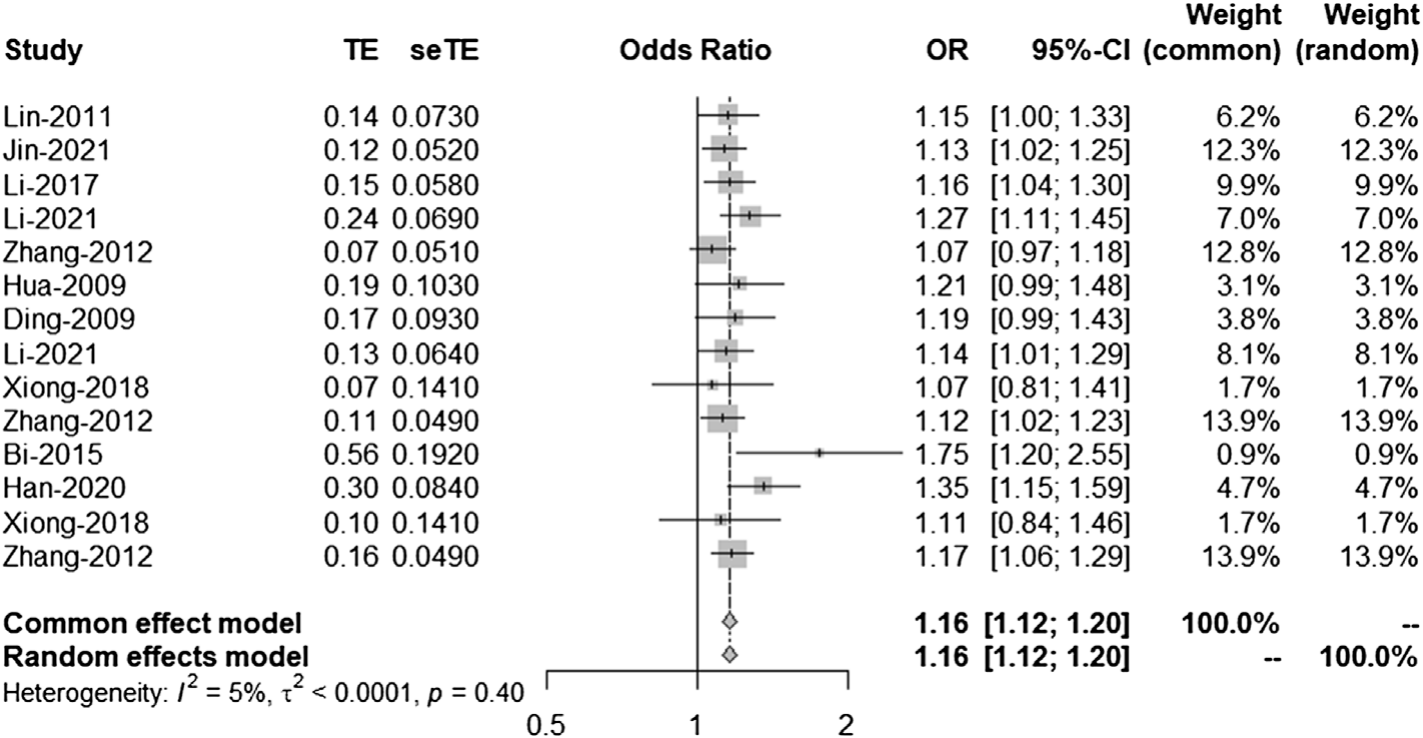
Fixed effects meta-analysis of the association between the three single-nucleotide polymorphisms (SNPs; rs2383207, rs2383206, and rs10757278) and ischemic stroke (IS) in the second analysis. CI, confidence interval; OR, odds ratios.

**FIGURE 5:**
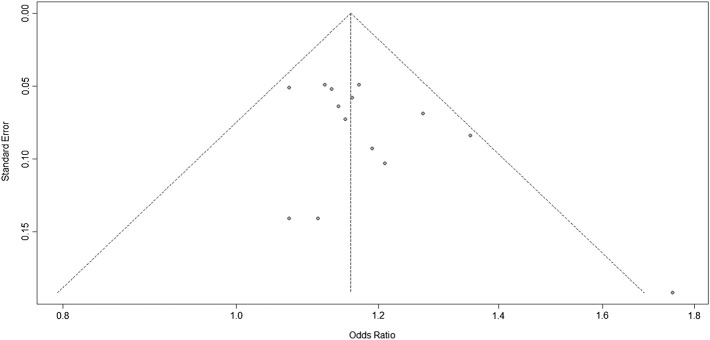
Funnel plots corresponding to the fixed-effects meta-analysis of the three single-nucleotide polymorphisms (SNPs; rs2383207, rs2383206, and rs10757278) and ischemic stroke (IS) in the second analysis.

According to the results of the bias test and sensitivity analysis, it was found that the studies by Yang et al. (rs2383207) (Yang et al., 2018) and Ding et al. (rs10757278) (Ding et al., 2009) had roughly the same weight and were outside the funnel plot. We decided to remove the two studies and re-analyze the results. After removing two studies, we used R program to re-analyze the remaining studies. In the second analysis, chromosome 9p21 remained significantly associated with IS risk, and the G allele was associated with increased IS risk (OR: 1.16, 95% CI: 1.12–1.20, *p* < 0.0001, Figure 4). The results of this second analysis further confirmed that the two removed studies had little influence on the initial results. Moreover, there was homogeneity between the studies (*I* ^2^ = 5%, *p* = 0.40), and the two experiments were outliers. A second bias test revealed that bias = 1.4217, *p* = 0.0696 (Figure 5). Sensitivity tests for the individual studies were again performed to ensure that no single study significantly affected the results of each single-locus sensitivity analysis.

**FIGURE 6:**
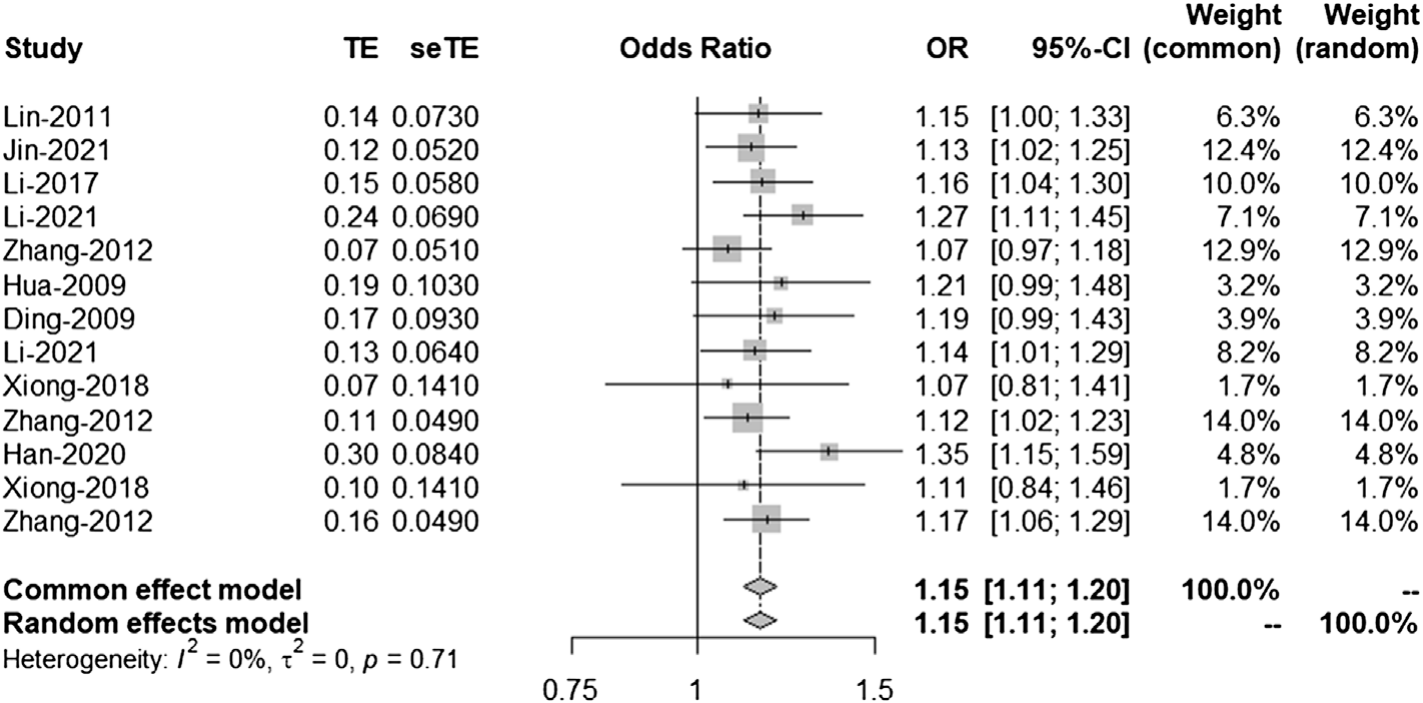
Fixed-effects meta-analysis of the associated between the three single-nucleotide polymorphisms (SNPs; rs2383207, rs2383206, and rs10757278) and ischemic stroke (IS) in the third analysis. CI, confidence interval; OR, odds ratios.

**FIGURE 7:**
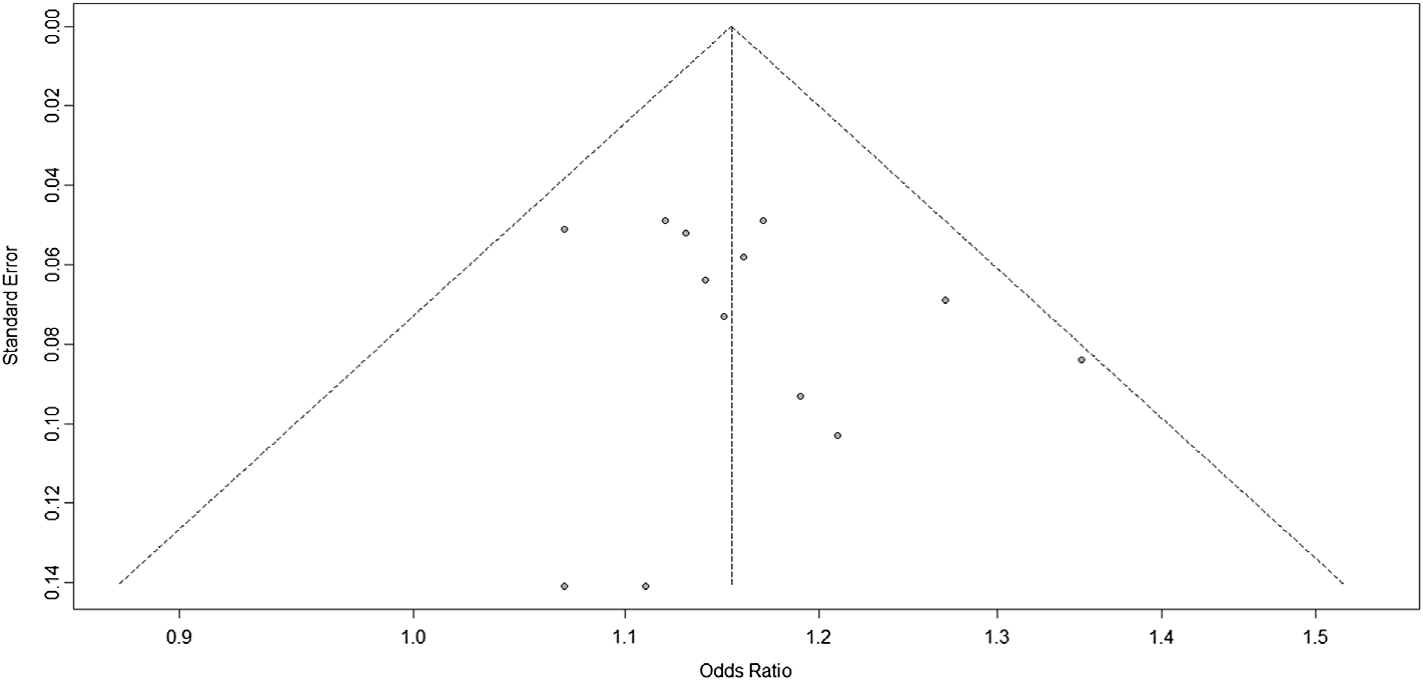
Funnel plots corresponding to the fixed-effects meta-analysis of the three single-nucleotide polymorphisms (SNPs; rs2383207, rs2383206, and rs10757278) and ischemic stroke (IS) in the third analysis.

We know from Figure 4 that the included studies were homogeneous, and the sensitivity analysis of each study also indicated that no single experiment significantly affected the experimental results. Therefore, based on the forest map and funnel plot, we also removed the study by Bi et al. (Bi et al., 2015), located outside the funnel plot, in the third analysis. In this third analysis, an increased risk of IS was associated with the G allele (OR: 1.16, 95% CI: 1.11–1.20, *p* < 0.0001, Figure 6). Further analysis confirmed that the homogeneity between studies was more significant after removing the study by Bi-2015 (*I* ^2^ = 0%, *p* = 0.71, Figure 7), and there was a more significant correlation between chromosome 9p21 and IS risk. Thus, the removal of the study by Bi et al. (2015) further verified our original conclusions. The experimental results indicate that chromosome 9p21 is significantly associated with IS risk, and an increased risk of IS is associated with the G allele.

## Discussion

Stroke is currently the main cause of death in China; it has high morbidity, mortality, and disability rates (Kim et al., 2015). Stroke can be clinically divided into two types: IS and hemorrhagic stroke. Among the stroke subtypes, hemorrhagic stroke accounts for 20–40% of strokes in Chinese population, while in most Western populations, the majority of strokes (80–90%) are cerebral infarctions (Reed, 1990). Furthermore, IS accounts for approximately 87% of all stroke types, and IS a multifactorial disease that is influenced by both genetic and environmental factors (Wang et al., 2021). Chromosome 921 was originally reported to be associated with coronary heart disease (Matarin et al., 2008). There are some similarities between the etiologies and mechanisms of coronary heart disease and stroke, and 9p21 variants are associated with both diseases (Matarin et al., 2008). However, when investigating the association between 9p21 and IS, the conclusions drawn by researchers in China and in the rest of the world have been inconsistent. Stroke is influenced by many factors, including genetic, environmental, and vascular risk factors. The main method of studying susceptibility sites and genes in complex diseases is GWAS, based on SNPs (McPherson et al., 2007).

Matthew Traylor et al. found that chromosome 9p21 and histone deacetylase were associated with stroke in individuals of European ancestry (Traylor et al., 2012). Furthermore, Akinyemi et al. reported that rs2383207 increases IS incidence in indigenous West African men (Akinyemi et al., 2017). Previously, GWAS was also used to demonstrate that the antisense non-coding RNA in the *INK4* locus (ANRIL) variants rs2383207 and rs1333049 increases the risk of IS and coronary heart disease in Caucasian populations (Dichgans et al., 2014; Dehghan et al., 2016). Notably, studies investigating the genetic associations of chromosome 9p21 variants have mainly been performed among Caucasian populations, and relatively few studies have been carried out in Han Chinese populations. Although Chen et al. (2019) studied chromosome 9p21 variants in Chinese populations, they concluded that mutations in rs2383207 may reduce the risk of IS but reported no definite correlation between rs10757278 and IS (Chen et al., 2019). In the present study, we once again focused on the relationship between stroke and chromosome 9p21.

In this meta-analysis, 18,584 participants were included; the IS and control groups contained 14,033 and 14,656 individuals, respectively. The three investigated SNPs have a linkage disequilibrium relationship, and we arrived at the same conclusions through unified analysis. All three SNPs were associated with IS risk. However, there was heterogeneity between the experimental results and studies; thus, bias detection and sensitivity analyses were carried out. Figure 3 suggested that the research may have been biased; therefore, to remove any possible bias, we performed another set of analyses. These further analyses had similar results that were more significant than those of the original analysis, further confirming that our analysis was correct.

In conclusion, our results indicate that rs2383206, rs2383207, and rs10757278 are significantly associated with IS risk and the G allele is associated with an increased risk of IS. Because the three SNPs in the present study have linkage disequilibrium and are in similar positions on chromosome 9p21, a unified analysis was performed. Environmental factors such as smoking and alcohol use may also be associated with IS risk, but not all studies considered these risk factors. Therefore, the influence of genes and the environment on IS pathogenesis needs to be further studied.

## Data Availability

The original contributions presented in the study are included in the article/Supplementary Material. Further inquiries can be directed to the corresponding author.
