# Corrigendum: Association of single-nucleotide polymorphisms of rs2383206, rs2383207, and rs10757278 with stroke risk in the Chinese population: A meta-analysis

**DOI:** 10.3389/fgene.2022.980218

**Published:** 2022-10-10

**Authors:** Xuemei Hu, Dongsen Wang, Chunying Cui, Qingjian Wu

**Affiliations:** ^1^ Clinical Medical College of Jining Medical University, Jining, China; ^2^ Department of Emergency, Jining No. 1 People’s Hospital, Jining, Shandong Province, China

**Keywords:** ischemic stroke, chromosome 9p21, rs2383206, rs2383207, rs10757278, Chinese

In the original article, there was an error. Incorrect information was published in the **abstract**.

A correction has been made to **Abstract**, **second sentence**:

“We systematically assessed the relationship between stroke and three 9p21 loci (rs2383206, rs2383207, and rs10757278) in this meta-analysis.”

The authors apologize for this error and state that this does not change the scientific conclusions of the article in any way. The original article has been updated.

